# Comprehensive 3D‐RISM analysis of the hydration of small molecule binding sites in ligand‐free protein structures

**DOI:** 10.1002/jcc.26406

**Published:** 2020-08-19

**Authors:** Takashi Yoshidome, Mitsunori Ikeguchi, Masateru Ohta

**Affiliations:** ^1^ Department of Applied Physics, Graduate School of Engineering Tohoku University Sendai Japan; ^2^ Drug Development Data Intelligence Platform Group, Medical Science Innovation Hub Program, Cluster of Science, Technology and Innovation Hub RIKEN Yokohama Japan; ^3^ Graduate School of Medical Life Science Yokohama City University Yokohama Japan

**Keywords:** distribution functions of water, hydration state, hydrogen bonds, ligand binding, statistical mechanical theory of solvation

## Abstract

Hydration is a critical factor in the ligand binding process. Herein, to examine the hydration states of ligand binding sites, the three‐dimensional distribution function for the water oxygen site, *g*_O_(***r***), is computed for 3,706 ligand‐free protein structures based on the corresponding small molecule–protein complexes using the 3D‐RISM theory. For crystallographic waters (CWs) close to the ligand, *g*_O_(***r***) reveals that several CWs are stabilized by interaction networks formed between the ligand, CW, and protein. Based on the *g*_O_(***r***) for the crystallographic binding pose of the ligand, hydrogen bond interactions are dominant in the highly hydrated regions while weak interactions such as CH‐O are dominant in the moderately hydrated regions. The polar heteroatoms of the ligand occupy the highly hydrated and moderately hydrated regions in the crystallographic (correct) and wrongly docked (incorrect) poses, respectively. Thus, the *g*_O_(***r***) of polar heteroatoms may be used to distinguish the correct binding poses.

## Graphical abstract

The hydration states of ligand binding sites are comprehensively analyzed using a theory of solvation. An analysis of the hydration states at the positions of ligand heavy atoms indicates that the polar heteroatoms of the ligand tend to occupy highly hydrated regions in the correct ligand poses and moderately hydrated regions in the incorrect ligand poses, suggesting a way to distinguish these two poses.

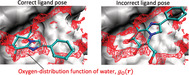

## 1 INTRODUCTION

Water molecules play important roles in ligand–protein binding.^1^ Upon ligand binding, the water molecules at the original binding site are displaced, and those close to the ligand also need to be rearranged. The free energy required for displacing the water molecules depends on their interaction with the protein, and this in turn greatly affects the binding free energy of the ligand. In addition, water‐mediated interactions between the ligand and protein often stabilize the ligand‐protein complex. Therefore, hydration at the binding site is essential for studying the binding of ligands to proteins.

The present investigation characterized the hydration states of small molecule (SM) binding sites in a large number of ligand‐free structures of SM–protein complexes. The ligand‐free structure was the protein part in the crystallographic structure of the SM–protein complex, obtained by simply removing the SM ligand structure and crystallographic waters (CWs). Meanwhile, metal ions such as zinc and crystallographic agents were considered a part of the protein and included in the ligand‐free structure.

The water molecules displaced upon ligand binding can be analyzed if the crystallographic structures of the protein‐ligand complex and the *apo* structure are both available.^2^ However, as this is not always the case, the hydration states of many proteins cannot be analyzed in this manner. Thus, it is necessary to analyze the hydration state of the binding site using computational methods. The implicit solvent model is not suitable for this purpose because of the difficulty in incorporating the effect of hydrogen bonding between the protein and water molecules and the effect of the excluded volume (EV), even though both are important in hydration. One reported approach for studying the hydration states of ligand‐free structures is the WaterMap.^3^, ^4^ In the WaterMap method, a molecular dynamics (MD) simulation is first performed for the ligand‐free structure in explicit solvent. The hydration sites are defined by 1 Å spheres that are very frequently occupied by water molecules, and then the free energy change upon releasing a water molecule at the hydration site is computed. However, the high computational cost of WaterMap hinders its application to a large number (e.g., thousands) of proteins. Another method is the WaterDock proposed by Ross et al.,^5^ which is a docking‐based method and uses AutoDock Vina to identify the water binding sites. The short computation time of WaterDock enabled the analysis of 14 proteins in their *holo* structures, and the water molecules were classified as “conserved” and “displaced” with 75% accuracy. Nevertheless, the scoring function implemented is empirical and lacks both the EV effect and the interactions between water molecules.

Here, we employed the three‐dimensional reference‐interaction site model (3D‐RISM) theory,^6^ which is a statistical mechanical theory of solvation, to analyze the hydration states of ligand‐free structures. This theory enabled us to compute the three‐dimensional water site distribution functions around a ligand‐free protein structure with the force fields used in MD simulations. The EV effect and the interactions between water molecules are explicitly included. The usefulness of the 3D‐RISM theory for analyzing hydration sites has been demonstrated before. For example, the positions of CWs inside the cavity of hen egg‐white lysozyme were successfully reproduced.^7^ In that case, the water binding sites are not exposed to the surface, and MD‐based methods such as WaterMap cannot be easily applied because the duration of the MD simulation is shorter than the time scale for the water molecules to penetrate the protein surface and reach the binding site inside. The other advantage of 3D‐RISM is that its computation time is much shorter than that of MD. Thus, this approach allows one to analyze the hydration states for thousands of ligand‐free structures.

In this study, the 3D‐RISM theory was used to compute the three‐dimensional distribution functions of water around the ligand‐free structures of 3706 proteins, which were obtained from the protein–ligand complexes in the PDBbind refined set (v. 2017).^8^, ^9^, ^10^, ^11^, ^12^, ^13^ The 3D‐RISM theory has been previously employed to calculate the hydration free energies for a large number of SMs^14^, ^15^, ^16^ and a large number of conformations of a single protein obtained through MD simulations.^17^, ^18^ However, to the best of our knowledge, its application to the hydration states of thousands of different proteins has not been reported.

## 2 METHODS

### 2.1 3D‐RISM theory

The three‐dimensional water site distribution function around the ligand‐free protein structures was computed using the 3D‐RISM theory implemented in the AmberTools18 suite.^19^ Here, we briefly outline the computation process, while the details can be found in reference books.^6^, ^20^ The process is split into two steps for treating the bulk water (step 1) and the aqueous solution of a ligand‐free protein structure at infinite dilution (step 2). In step 1, the water site‐site correlation functions were computed with the dielectrically consistent RISM (DRISM) theory^21^ combined with the Kovalenko‐Hirata (KH) closure.^22^ Then, in step 2, the three‐dimensional water site distribution function around the ligand‐free protein was obtained from the 3D‐RISM theory, using the water site‐site correlation functions from step 1 as input and the KH closure. For the water site *α* = H (hydrogen) or O (oxygen), the distribution function at position ***r*** is denoted by *g*_α_(***r***). Here, the analysis was carried out using *g*_O_(***r***).

As described in the introduction, the interactions between water molecules as well as the interactions between water molecules and protein atoms are explicitly treated in the 3D‐RISM theory. Owing to the interactions between water molecules, *g*_O_(***r***) has multiple peaks.^6^, ^20^ While the peak nearest to the protein surface primarily arises from the interactions between water molecules and protein atoms, the other peaks primarily arise from the interactions between water molecules.

The following force fields and parameters were used for the calculations with the 3D‐RISM theory. Amber ff99SB^23^ was used for the proteins and ions, while the coincident SPC/E model^24^ was employed for water. The histidine residue was set with a hydrogen on the delta nitrogen, HID. In step 1, the values of the dielectric constant and the bulk density were set at 78.497 and 0.03332 Å^−3^, respectively. The temperature was set at 310 K. The other parameters required for the computation were set at the default values implemented in the AmberTools18 suite. In step 2, a water box was prepared, and its size was set so that the minimum distance between the protein and the edge of the box was 14 Å. The linear grid spacings for the *x*, *y*, and *z* coordinates were set to 0.5 Å, and the maximum number of steps for convergence was 20,000. The default values implemented in the AmberTools18 suite were employed for the other parameters.

### 2.2 Data set

Protein–ligand complexes in the PDBbind refined set (v. 2017)^8^, ^9^, ^10^, ^11^, ^12^, ^13^ consisting of 4,154 complexes were utilized as the data set for analysis. In the PDBbind data set, hydrogen atoms were already added to the heavy atoms other than those of the CWs. For each complex, the ligand and the CWs were removed, and the remainder (proteins, ions, and other ligands such as crystallization agents) was used in the following 3D‐RISM calculation as a ligand‐free structure. The ligand was separately saved in the Tripos Mol2 format. When the crystallographic protein structure contained multiple protein chains, the chain closest to the ligand was selected. Hereinafter, the resulting molecules are simply referred to as “proteins.”

Before the 3D‐RISM calculations, the following preprocessing was applied to the proteins. First, CAPs (the acetyl group and *N*‐methylamide, denoted respectively by ACE and NME in Amber) were added to the N‐ and C‐terminals. ACE and NME were added to the residues before and after any missing fragments. For example, in chain H of thrombin (PDB code: 1a4w), residues 147–149 were missing. In this example, ACE and NME were added to the C‐terminal side of Glu146 and to the N‐terminal side of Gly150, respectively. Namely, residues 147 and 149 were ACE and NME, respectively. The same treatment was performed for the missing fragments in the other proteins. Second, the *tleap* command in the AmberTools18 suite was executed to assign the force field parameters for each atom. Proteins that produced errors under this command were excluded from the subsequent calculations. Then, a minimization of the protein structure was performed using the AmberTools18 suite with the constraint of 10.0 kcal mol^−1^ Å^−2^ for the heavy atoms to optimize the positions of the hydrogen atoms. During the minimization, the generalized Born model was employed for the solvent. Again, proteins that produced any error during the minimization were excluded. Finally, a total of 3,706 proteins were used for the subsequent computations with the 3D‐RISM theory.

### 2.3 Analysis of water oxygen distribution function with crystallographic waters

To analyze the distribution of solvent water, the distribution function *g*_O_(***r***) at the position of the CWs was examined. To focus on the CWs near the protein surface, only those having at least one protein heavy atom within 5.0 Å of the surface were selected. The 2,403 proteins having CWs in their PDB data were used for the analysis, and the total number of the CWs was approximately 620,000.

To assess the hydration states at the experimentally determined CW positions, the *g*_O_(***r***) values calculated by the 3D‐RISM theory were used to determine the distribution function at the position of each CW (*g*_O_(***r***_CW_), where ***r***_CW_ represents the position of the CW) as follows. Note that ***r***_CW_ was not always on the grid points where *g*_O_(***r***) was calculated. While one may simply use the *g*_O_(***r***) value at the nearest grid point for *g*_O_(***r***_CW_), in some cases this leads to *g*_O_(***r***_CW_) ≈ 0 because the nearest grid point can be inside the protein. To overcome this issue, *g*_O_(***r***_CW_) was chosen to be the maximum *g*_O_(***r***) value at the grid points within 0.9 Å of the CW. A larger *g*_O_(***r***_CW_) value means a higher probability that a water exists at ***r***_CW_. Thus, the *g*_O_(***r***_CW_) values are expected to be larger than 1 because *g*_O_ = 1 is the probability of oxygen in bulk water.

Next, the number of contacts with the protein heavy atoms was counted for each CW in order to discuss the characteristics of the *g*_O_(***r***_CW_) values. Such contacts were defined by a distance of 3.9 Å or less between the CW and the protein heavy atom, using the threshold value from the program HBPLUS.^25^ To analyze the elements for the protein heavy atoms in contact with the CWs, the minimum distance from the protein heavy atom to the CW, denoted by $r_{\mathrm{CW}\text{\_}P}^{\mathrm{Min}}$, was obtained for each CW. The element of this closest protein heavy atom (nitrogen, oxygen, carbon, sulfur, etc.) was used to investigate the relationships between *g*_O_(***r***_CW_) and the contacted elements on the protein, which were visualized using pie charts.

### 2.4 Analysis of water oxygen distribution function with ligand heavy atoms

For each ligand heavy atom (LHA) located at ***r***_LHA_, the distribution function of water oxygen at its position, *g*_O_(***r***_LHA_), was computed. For background information, *ρg*_O_(***r***_LHA_)Δ*V* (where *ρ* is the density of the bulk solvent) represents the number of water molecules within a small volume Δ*V* around ***r***_LHA_.^20^ Thus, a higher *g*_O_(***r***_LHA_) value indicates that more water molecules at ***r***_LHA_ are replaced upon ligand binding. *g*_O_(***r***_LHA_) was computed using the same procedure as for *g*_O_(***r***_CW_). During this calculation, the LHAs were categorized according to the SYBYL atom types, which were obtained using the information listed in the ligand file in the Tripos Mol2 format. The SYBYL atom types are given in Table 1, where those shown in boldface were the focus of this study because there were enough data points for the analysis.

**TABLE 1:** List of SYBYL atom types

Atom type	Notation
Hydrogen	H
Carbon sp3	**C.3**
Carbon sp2	**C.2**
Carbon sp	C.1
Carbon aromatic	**C.ar**
Carbocation	**C.cat**
Nitrogen sp3	N.3
Nitrogen sp2	**N.2**
Nitrogen sp	N.1
Nitrogen aromatic	**N.ar**
Nitrogen amide	**N.am**
Nitrogen trigonal planar	**N.pl3**
Nitrogen sp3 positively charged	**N.4**
Oxygen sp3	**O.3**
Oxygen sp2	**O.2**
Oxygen in carboxylates and phosphates	O.co2
Sulfur sp3	**S.3**
Sulfur sp2	S.2
Sulfoxide sulfur	S.o
Sulfone sulfur	**S.o2**
Phosphorus sp3	**P.3**
Fluorine	**F**
Chlorine	**Cl**
Other halogens and metals	—

*Note:* The probability of *g*_O_(***r***) was analyzed for the atom types shown in boldface.

To investigate the elements of the protein heavy atoms in contact with the ligands, the minimum distance from the protein heavy atom to each LHA, denoted by $r_{\mathrm{LHA}\text{\_}P}^{\mathrm{Min}}$, was defined. For each LHA with a *g*_O_(***r***_LHA_) value, the element of the closest protein heavy atom at $r_{\mathrm{LHA}\text{\_}P}^{\mathrm{Min}}$ was assigned and visualized using a pie chart.

Two ligand poses were considered for each protein: a correct one and an incorrect one. The former was based on the crystallographic ligand structure (converted to the Tripos Mol2 format in the PDBbind database). The latter was selected from the ligand poses generated using AutoDock Vina for each of the 3,706 proteins.^26^ The following process was used to select a pose that was dissimilar to the correct pose. First, the binding poses for which the root‐mean‐square deviation (RMSD) with the correct pose was between 4.5 and 5.5 Å were selected. The poses with RMSD values less than 4.5 Å were excluded because they are too similar to the correct pose. Those with RMSD values larger than 5.5 Å were also excluded to avoid the possibility of the ligand being outside the binding pocket. Next, if multiple poses had RMSD values between 4.5 and 5.5 Å, the binding pose with RMSD value nearest to 5 Å was selected as the “incorrect pose.” If no binding pose had an RMSD value between 4.5 and 5.5 Å, the corresponding PDBbind entry was not used for the analysis of LHAs.

### 2.5 Definitions of terms

In the subsequent sections, the terms “highly hydrated” and “moderately hydrated” are used. A CW, region, state, or binding site that is “highly hydrated” is defined by *g*_O_(***r***) > 4, whereas one that is “moderately hydrated” is defined by 1 < *g*_O_(***r***) < 4.

## 3 RESULTS AND DISCUSSION

### 3.1 Analysis of crystallographic waters with ***g***_**O**_(***r***)

**FIGURE 1:**
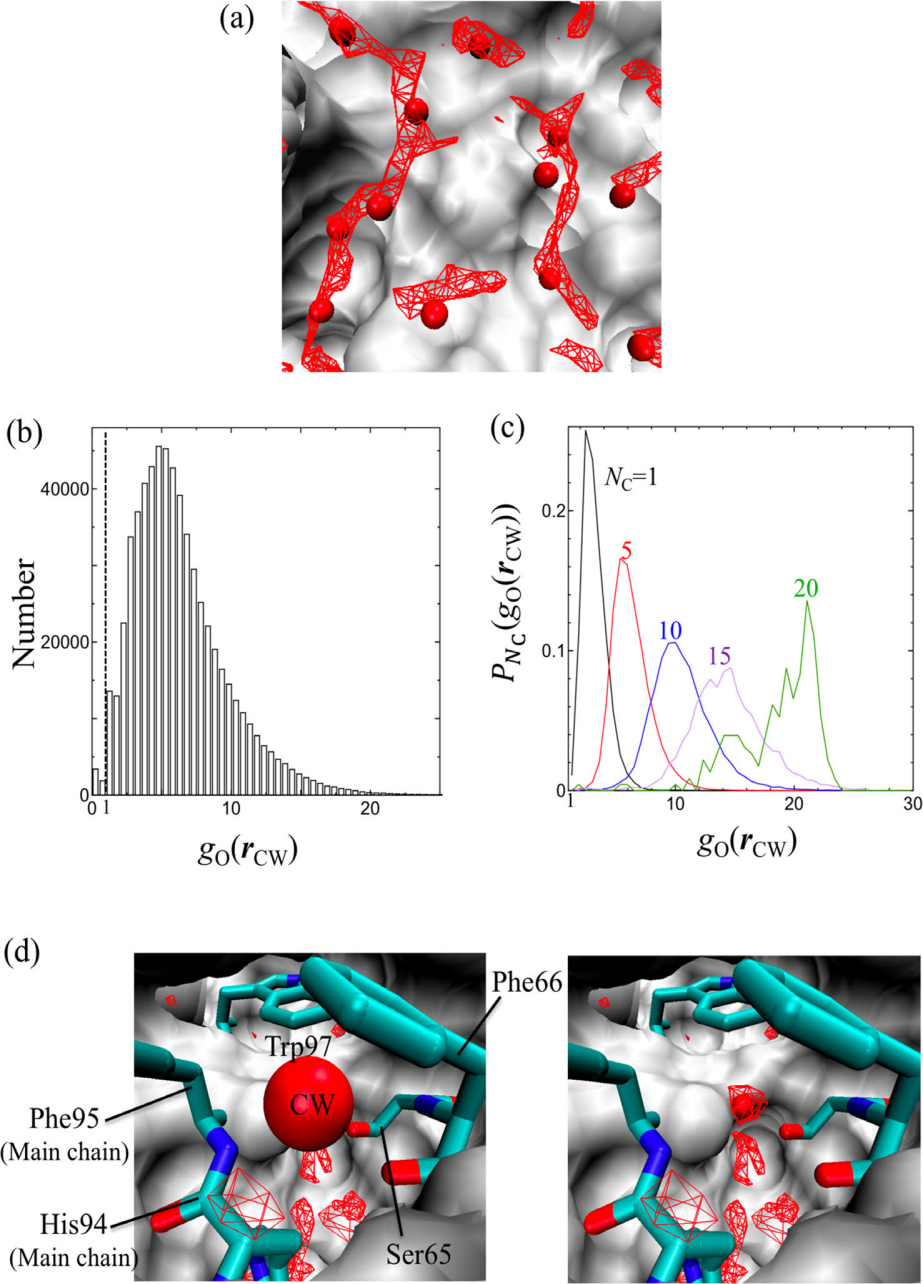
(a) Three‐dimensional distribution function of the oxygen site of water, *g*_O_(***r***), around dihydrofolate reductase (PDB code: 1dhi). Spheres: crystallographic waters (CWs). Red mesh: regions with *g*_O_(***r***) > 4. Surface: protein. VMD software was used for the visualization.^27^ (b) Histogram of *g*_O_(***r***_CW_). The dotted line represents *g*_O_(***r***_CW_) = 1. (c) Probability of *g*_O_(***r***_CW_), $P_{N_{C}}\left[g_{O}\left(r_{\mathrm{CW}}\right)\right]$, for different *N*_C_ values. Contact is defined based on the distance between the CW and the heavy atoms in the protein. (d) Example CW with *N*_C_ = 20 (*g*_O_(***r***_CW_) = 14.4) for human carbonic anhydrase I enzyme (PDB code: 1azm). Residues in contact with the CW are shown in licorice representation. Red mesh: regions with *g*_O_(***r***) > 6 [Color figure can be viewed at wileyonlinelibrary.com]

To check the results of our 3D‐RISM calculations, the overlap between *g*_O_(***r***) and ***r***_CW_ for dihydrofolate reductase (PDB code: 1dhi) was examined visually in Figure 1a. Regions where *g*_O_(***r***) ≥ 4.0 (colored in red) strongly overlap with the CWs. In the red regions, the probability for the existence of solvent water molecules is at least four times higher than that in bulk water. Thus, the observation of CWs in the red regions is reasonable, and it indicates that the 3D‐RISM theory successfully reproduced the positions of these CWs. Figure 1a also shows that the regions with high *g*_O_(***r***) are tube‐shaped, suggesting that the movement of water molecules is easy within the tube but relatively hard outside of it.

The distribution function of water molecules at each CW, *g*_O_(***r***_CW_), was computed for the comprehensive analysis of the hydration state at the CWs. In the histogram in Figure 1b, *g*_O_(***r***_CW_) > 1 at most of the CW positions. Therefore, these CWs are located in regions with a higher probability for the existence of water than that in the bulk, which is also a reasonable result. It was observed that the histogram has a maximum at *g*_O_(***r***_CW_) ∼ 5, and the range of *g*_O_(***r***_CW_) exceeds 30. Regarding the hydration states, the peak at *g*_O_(***r***_CW_) ∼ 5 means that the hydration states with these *g*_O_(***r***_CW_) values are most frequently distributed around the proteins, based on the comprehensive calculation for 3,706 proteins using the 3D‐RISM theory.

We also counted the number of contacts with the protein heavy atoms, *N*_C_, so as to understand the value of *g*_O_(***r***_CW_) for each CW at the atomic level. Then, the probability of *g*_O_(***r***_CW_) for the CWs with *N*_C_ contacts, $P_{N_{C}}\left[g_{O}\left(r_{\mathrm{CW}}\right)\right]$, was obtained. Figure 1c shows $P_{N_{C}}\left(g_{O}\left(r_{\mathrm{CW}}\right)\right)$ for *N*_C_= 1, 5, 10, 15, and 20. Clearly, the highest peak of $P_{N_{C}}\left[g_{O}\left(r_{\mathrm{CW}}\right)\right]$ is strongly correlated with *N*_C_: the position of this peak is shifted toward the right (larger *g*_O_(***r***_CW_)) when *N*_C_ is increased. Thus, a region with very high *g*_O_ will appear as almost completely surrounded by the protein. Figure 1d shows an example CW with *N*_C_= 20 (*g*_O_(***r***_CW_)= 14.4), which is surrounded by Ser65, Phe66, His94, Phe95, and Trp97, and half of its contacts are formed with the heavy atoms in Phe66.

**FIGURE 2:**
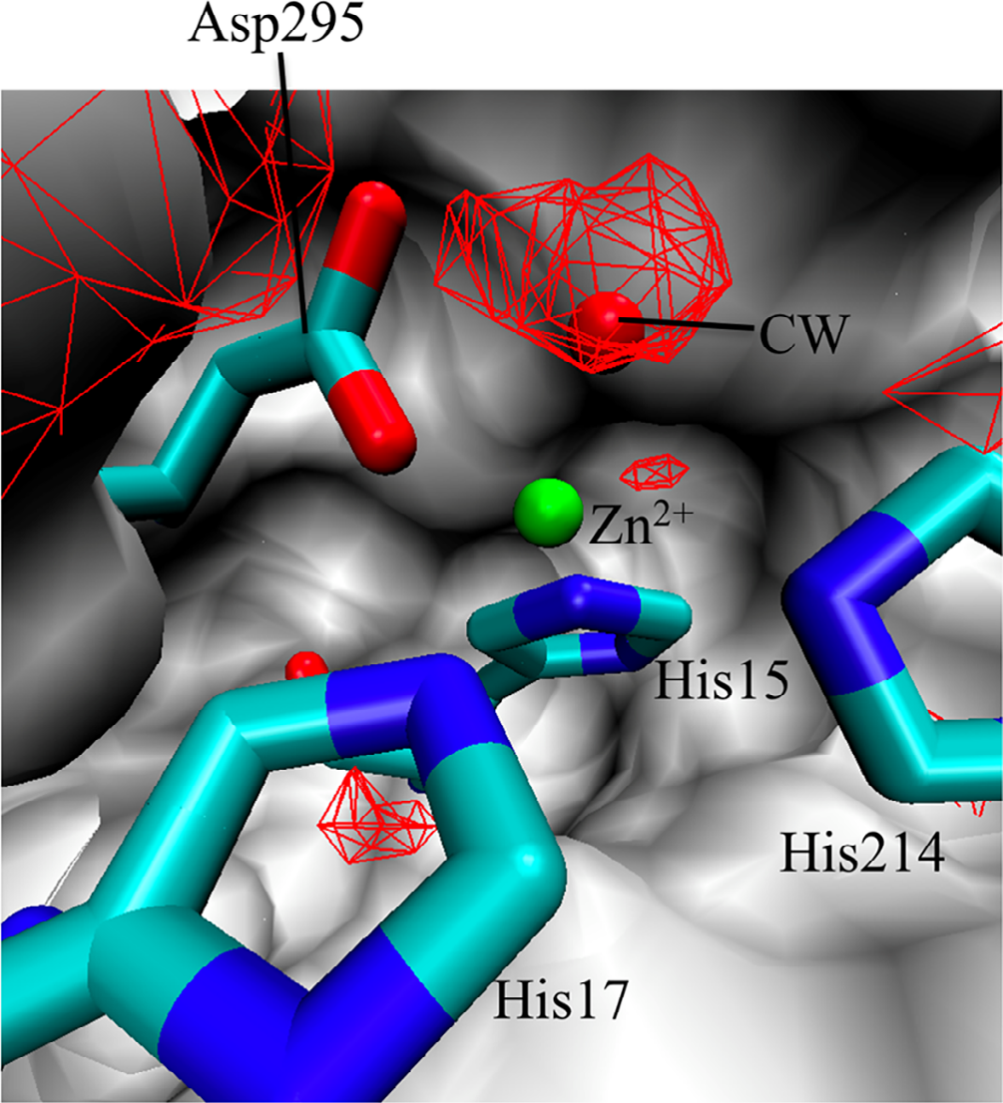
CW with *g*_O_(***r***_CW_) ≥ 30 (red sphere) for adenosine deaminase (PDB code: 1add). Surface: protein. Red mesh: regions with *g*_O_(***r***) > 4 [Color figure can be viewed at wileyonlinelibrary.com]

As described earlier, the range of *g*_O_(***r***_CW_) in the histogram of Figure 1b exceeds 30. Most of the CWs with *g*_O_(***r***_CW_) ≥ 30 were very close to ions. One example is depicted in Figure 2. In this case, the CW was coordinated to Zn^2+^, and the electrostatic interactions between the ion and water molecules led to very high *g*_O_(***r***_CW_) values. Another case of *g*_O_(***r***_CW_) ≥ 30 is the situation shown in Figure 1d, where the CW is deeply buried inside the protein.

### 3.2 Analysis of hydration states close to ligands

**FIGURE 3:**
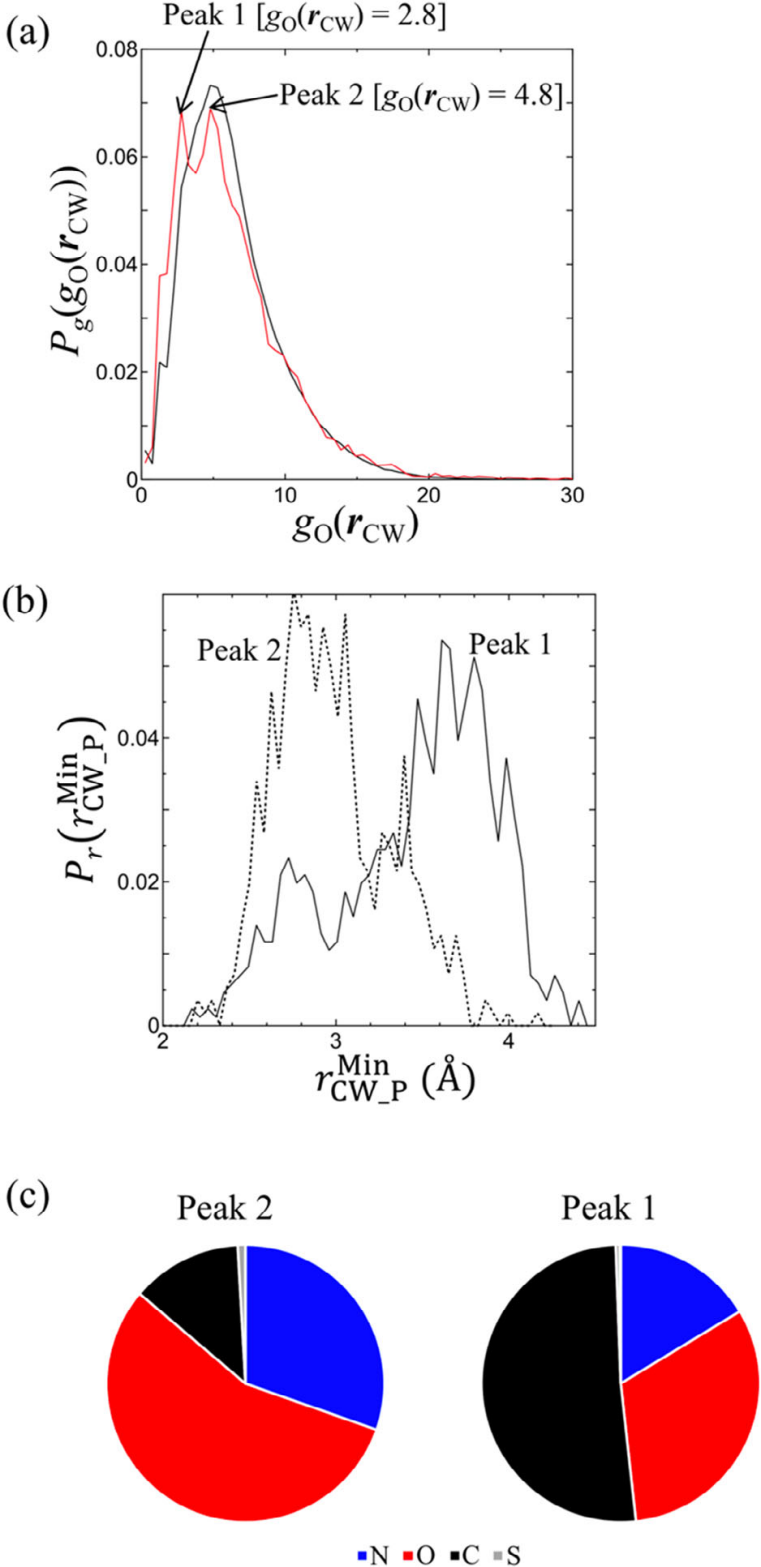
(a) Probabilities of *g*_O_(***r***_CW_). The black line corresponds to all the CWs and is the same as the histogram in Figure 1b, except that the histogram is normalized to 1. The red line corresponds to the CWs close to the ligand. (b) Probabilities of *r*_min,P_ for the CWs with *g*_O_(***r***_CW_) = 2.8 (peak 1) and 4.8 (peak 2). (c) Pie charts for elements of the protein heavy atoms at $r_{\mathrm{CW}\text{\_}P}^{\mathrm{Min}}$ interacting with the CWs corresponding to peaks 1 and 2 [Color figure can be viewed at wileyonlinelibrary.com]

To investigate the effects of ligands on *g*_O_(***r***_CW_), we analyzed *g*_O_(***r***_CW_) at CW positions that are at most 4.0 Å away from any of the LHAs. The number of these CWs is approximately 45,000. In Figure 3a, the histogram of *g*_O_(***r***_CW_) for these CWs close to the ligand (red line) is compared to that for all CWs (black line). Both histograms are normalized to one because they contain different numbers of CWs. The normalized probability (*Y*‐axis) is denoted by *P*_*g*_(*g*_O_(***r***_CW_)) hereinafter. While the shapes of both *P*_*g*_(*g*_O_(***r***_CW_)) profiles were almost the same for *g*_O_(***r***_CW_) ≥ 10, when *g*_O_(***r***_CW_) < 10, the probabilities appeared very different for CWs close to the ligands and all CWs. Only one peak at *g*_O_(***r***_CW_)∼4.8 was observed for all CWs (black line), while there were two peaks for the CWs closest to the ligands (red line) at *g*_O_(***r***_CW_)∼2.8 and ∼4.8, and the height of the latter peak was slightly decreased compared to its counterpart for all CWs.

To investigate the atomic origin of the two peaks in *P*_*g*_(*g*_O_(***r***_CW_)) for CWs close to the ligands, the minimum distance from the protein heavy atom to the CW, $r_{\mathrm{CW}\text{\_}P}^{\mathrm{Min}}$, was computed for each CW involved in either peak. The probabilities of $r_{\mathrm{CW}\text{\_}P}^{\mathrm{Min}}$, hereinafter denoted by $P_{r}\left(r_{\mathrm{CW}\text{\_}P}^{\mathrm{Min}}\right)$, are shown in Figure 3b for the CWs with *g*_O_(***r***_CW_) = 2.8 (peak 1) and 4.8 (peak 2). The $P_{r}\left(r_{\mathrm{CW}\text{\_}P}^{\mathrm{Min}}\right)$ profiles were significantly different. For peak 1 (black solid line), $P_{r}\left(r_{\mathrm{CW}\text{\_}P}^{\mathrm{Min}}\right)$ had two peaks at $r_{\mathrm{CW}\text{\_}P}^{\mathrm{Min}}∼$ 2.8 and 3.6 Å, with the latter being the major peak. For peak 2 (black dashed line), there was no peak in $P_{r}\left(r_{\mathrm{CW}\text{\_}P}^{\mathrm{Min}}\right)$ at $r_{\mathrm{CW}\text{\_}P}^{\mathrm{Min}}∼$ 3.6 Å, and the height of the major peak at $r_{\mathrm{CW}\text{\_}P}^{\mathrm{Min}}∼$ 2.8 Å was substantially increased.

The CWs belonging to peaks 1 and 2 differed in the type of nearest protein heavy atom elements (Figure 3c). For the CWs with *g*_O_(***r***_CW_) = 4.8 (peak 2), the nearest‐neighbor protein heavy atoms were mainly nitrogen and oxygen, while for half of the CWs with *g*_O_(***r***_CW_) = 2.8 (peak 1), the nearest‐neighbor protein heavy atoms were carbon.

**FIGURE 4:**
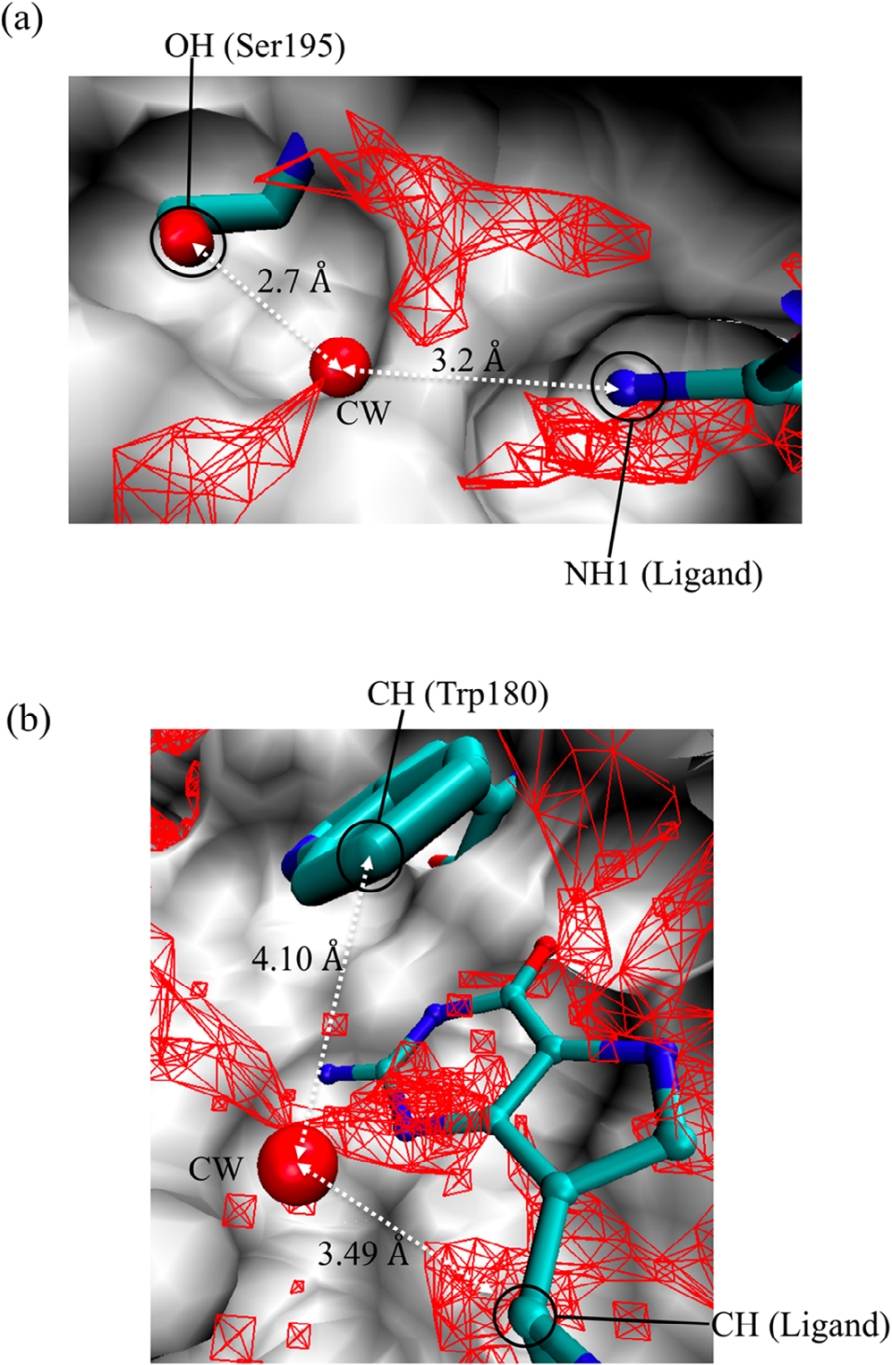
CWs (shown as spheres) corresponding to (a) peak 2 of thrombin (PDB code: 1a4w) and (b) peak 1 of *Giardia* GPRTase (PDB code: 1dqn). Surface: protein. Red mesh: regions with (a) *g*_O_(***r***) > 3.8 and (b) *g*_O_(***r***) = 2.3–3.0. The ligands (QWE of 1a4w and IMU of 1dqn) are shown in ball and stick representation. The residues interacting with the CW at the distance $r_{\mathrm{CW}\text{\_}P}^{\mathrm{Min}}$ are shown in licorice representation [Color figure can be viewed at wileyonlinelibrary.com]

From the results described above, the atomic‐level origin of the two peaks in *P*_*g*_(*g*_O_(***r***_CW_)) was given as follows. First, it was suggested the CWs belonging to peak 2 formed hydrogen bonds with the nitrogen and oxygen atoms of the proteins because these CWs had a maximum $P_{r}\left(r_{\mathrm{CW}\text{\_}P}^{\mathrm{Min}}\right)$ at $r_{\mathrm{CW}\text{\_}P}^{\mathrm{Min}}∼$ 2.8 Å (a typical distance for hydrogen bonds such as NH···O=C and OH···O=C), and the nearest‐neighbor protein heavy atom was mainly nitrogen and oxygen. An example is shown in Figure 4a, where the CW was stabilized by the hydrogen bond between it and the hydroxy group of Ser195. Another hydrogen bond was also formed between the CW and the amino group of the ligand. The resultant hydrogen‐bonding network of Ser195, the CW, and the ligand is thought to increase the stability of the CW.

For the CWs with *g*_O_(***r***_CW_) = 2.8 (peak 1), the major peak of $P_{r}\left(r_{\mathrm{CW}\text{\_}P}^{\mathrm{Min}}\right)$ was at $r_{\mathrm{CW}\text{\_}P}^{\mathrm{Min}}∼$ 3.6 Å, which is a typical distance for weak interactions such as the CH—O interaction, and half of the nearest‐neighbor protein heavy atoms were carbon. These findings suggested the formation of weak interactions such as CH—O between the CW and the protein atoms. For example, in Figure 4b a CH—O interaction was formed between the CH of Trp180 and the CW oxygen. The CW oxygen also forms another CH—O interaction with the CH of the ligand. In this case, the formation of a CH‐O network with Trp180, the CW, and the ligand is thought to help stabilize the CW. Thus, in both Figure 4a and b, an interaction network is formed between the protein, the CW, and the ligand to stabilize the CW. Such protein–CW–ligand networks are believed to result from water rearrangement after ligand binding. Overall, there seems to be two types of protein–CW–ligand interaction networks: hydrogen‐bonding networks derived from highly hydrated CWs and weak CH—O interaction networks originating from moderately hydrated CWs. These interaction networks induced by the ligand might be a key factor in the formation of ligand/water/protein complexes.

### 3.3 Analysis of hydration states at ligand heavy atoms

**FIGURE 5:**
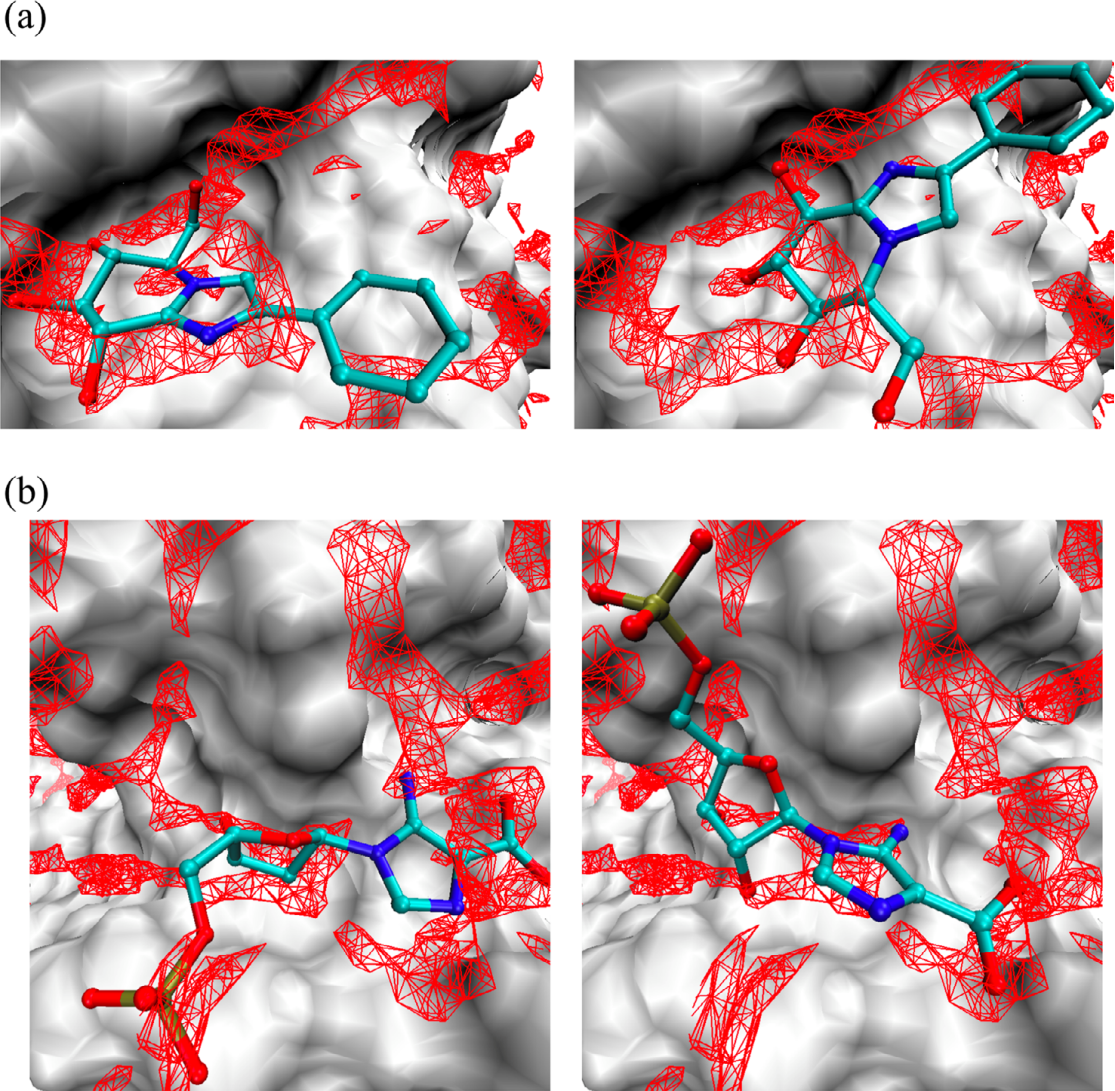
Three‐dimensional distribution functions of the oxygen site of water, *g*_O_(***r***), around (a) β‐D‐glucan glucohydrolase (PDB code: 1x38) and N5‐carboxyaminoimidazole ribonucleotide mutase (PDB code: 2nsl). Left: correct binding pose. Right: incorrect binding pose. The ligands (IDO of 1x38 and C2R of 2nsl) are shown in ball and stick representation. Red mesh: regions with (a) *g*_O_(***r***) > 3.7 and (b) *g*_O_(***r***) > 3.5. Surface: protein. To clearly show the distribution function and the ligand, the residues between Gly429‐Thr438 and Val484‐Gly494 are ignored in (a), and those between Ala44‐Arg46 and Ala73‐His75 are ignored in (b) [Color figure can be viewed at wileyonlinelibrary.com]

To examine the characteristics of water molecules to be replaced by the ligand at the protein binding site, *g*_O_(***r***_LHA_) was examined for the correct and incorrect poses of the ligand. Figure 5 shows the results for β‐D‐glucan glucohydrolase (PDB code: 1x38) and N5‐carboxyaminoimidazole ribonucleotide mutase (PDB code: 2nsl). Apparently, the degree of overlap between the regions with high *g*_O_(***r***) (red mesh) and the LHAs was significantly different between the two poses. In the correct pose for β‐D‐glucan glucohydrolase (Figure 5a, left), all the polar heteroatoms in the ligand, which are the oxygen atoms of the hydroxy groups and nitrogen atoms of the glucoimidazole ring, overlapped well with the red mesh. In the incorrect pose (Figure 5a, right), although the oxygen atoms of the LHAs overlapped with the red mesh, this was not the case for any of the nitrogen atoms in the glucoimidazole ring. Similar behavior was observed for N5‐carboxyaminoimidazole ribonucleotide mutase. In the correct crystallographic binding pose (Figure 5b, left), most of the polar heteroatoms overlapped well with the red mesh, whereas in the incorrect binding pose (Figure 5b, right), the overlap was not as extensive for the polar heteroatoms of the LHAs. Thus, in both examples, the regions with high *g*_O_(***r***) (as depicted by red mesh) were replaced by the polar heteroatoms of the ligand in the correct pose but not in the incorrect pose.

**FIGURE 6:**
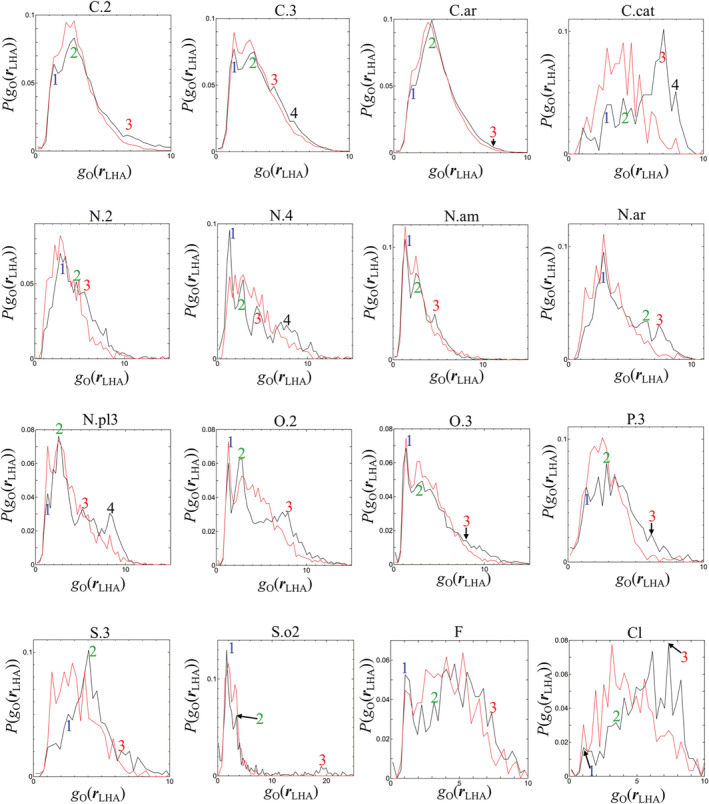
Probabilities of *g*_O_(***r***_LHA_) for the ligand atom types shown in boldface in Table 1. Black: correct pose. Red: incorrect pose. The numbered points are analyzed in Figure 7 [Color figure can be viewed at wileyonlinelibrary.com]

To comprehensively discuss the overlap between *g*_O_(***r***) and LHAs, the probabilities of *g*_O_(***r***_LHA_), i.e., P(*g*_O_(***r***_LHA_)), were examined for the SYBYL atom types shown in boldface in Table 1 (Figure 6). The data for regions with high *g*_O_(***r***_LHA_) values are illustrated in Figure S1 in the Supporting Information. For sp2 carbon (C.2), sp3 carbon (C.3), and aromatic carbon (C.ar), although P(*g*_O_(***r***_LHA_)) in the highly hydrated range (*g*_O_(***r***_LHA_)≥ 4) was only slightly higher for the correct pose (black) than for the incorrect pose (red), the shape for each pose was almost the same.

For some other atom types, these characteristics were different between the poses. Figure 6 shows that the value of P(*g*_O_(***r***_LHA_)) at high *g*_O_(***r***_LHA_) (highly hydrated states) was higher for the correct poses (black line) than for the incorrect poses (red line). For example, for sp2 oxygen (O.2) such as carbonyl oxygen, three peaks were observed at *g*_O_(***r***_LHA_)= 1.35, 2.85, and 7.95 for the correct pose (the positions of these peaks are denoted by points 1, 2, and 3, respectively, in Figure 6). At point 3 (*g*_O_(***r***_LHA_)= 7.95, that is, highly hydrated states), P(*g*_O_(***r***_LHA_)) was higher for the correct pose than for the incorrect one. In contrast, the peak of P(*g*_O_(***r***_LHA_)) near point 1 (*g*_O_(***r***_LHA_)= 1.35, that is, moderately hydrated states) was higher for the incorrect pose. This was also true for the following atom types (Here “moderately hydrated” and “highly hydrated” are denoted by “MH” and “HH”, respectively. Although most of the selected points correspond to the positions of peaks in Figure 6, some points were selected at non‐peak positions to allow comparisons between the P(*g*_O_(***r***_LHA_)) profiles for the HH and MH regions. Hereinafter, all selected positions are referred to using “point”.):

- sp2 nitrogen (N.2), MH point 1 vs. HH point 3
- Cationic nitrogen (N.4), MH point 2 vs. HH point 4
- Amide nitrogen (N.am), MH point 1 vs. HH point 3
- Aromatic nitrogen (N.ar), MH point 1 vs. HH point 3
- Planar sp3 nitrogen (N.pl3), MH point 1 vs. HH point 4
- sp3 phosphorus (P.3), MH point 2 vs. HH point 3
- sp3 sulfur (S.3), MH point 1 vs. HH point 3
- Fluorine (F), MH point 2 vs. HH point 3
- Chlorine (Cl), MH point 1 vs. HH point 3

The present results strongly suggested that the correct and incorrect poses might be distinguished based on the *g*_O_(***r***_LHA_) of heteroatoms, that is, the ligand pose with larger *g*_O_(***r***_LHA_) values for the heteroatoms might be the correct binding pose. The present analysis was feasible because *g*_O_(***r***) was calculated using the 3D‐RISM theory, in which the computation for each ligand‐free structure took less than 2 hr. In comparison, such a comprehensive large‐scale analysis of hydration would be challenging using the WaterMap method because the MD simulation employed there would require very heavy calculation.

### 3.4 Analysis of binding site hydration and ligand–protein interactions

**FIGURE 7:**
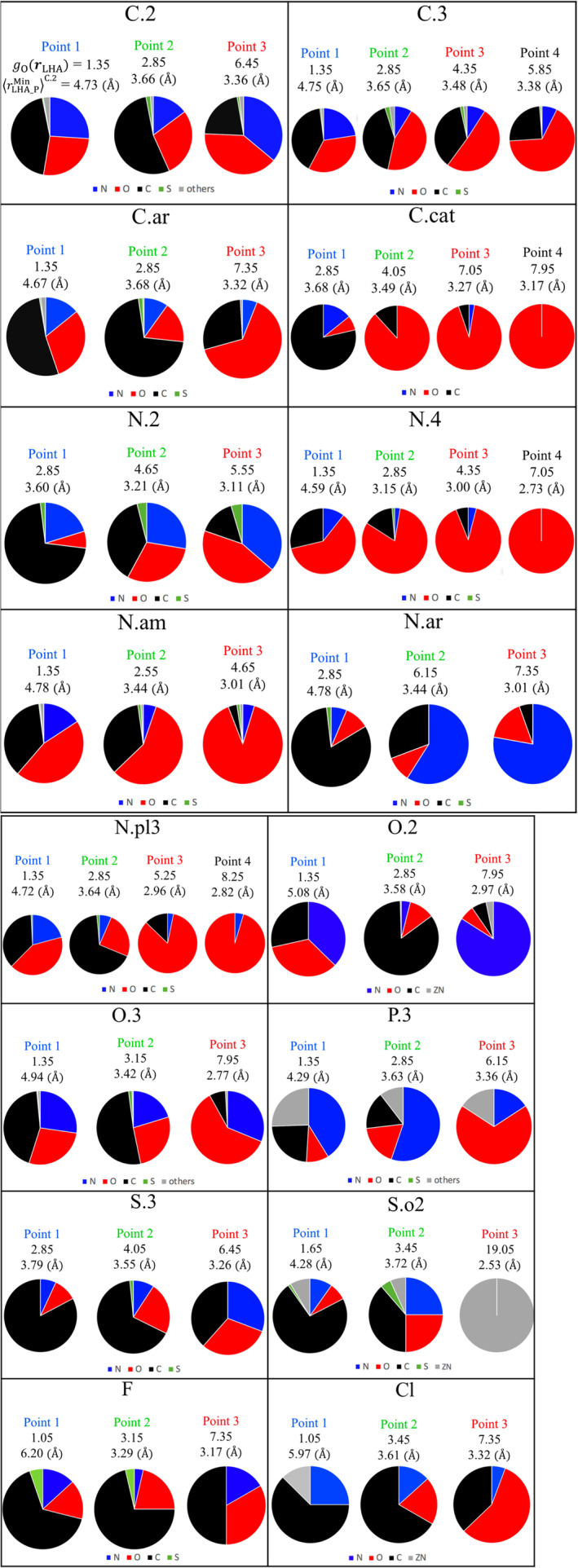
Pie charts for the ligand atom types shown in boldface in Table 1. The point number, *g*_O_(***r***_LHA_), and $r_{\mathrm{LHA}\text{\_}P}^{\mathrm{Min}}$ are shown in each chart [Color figure can be viewed at wileyonlinelibrary.com]

To further investigate the selected points on *g*_O_(***r***_LHA_) shown in Figure 6, which as previously mentioned correspond to the peaks in the correct poses as well as some other points in the highly hydrated regions, we analyzed the detailed origin of the ligand‐protein and water‐protein interactions. The average of the minimum distance from the protein heavy atom to each LHA, $r_{\mathrm{LHA}\text{\_}P}^{\mathrm{Min}}$, for all the LHAs of atom type X in the ligand at point *i* in Figure 6 was defined as ${〈r_{\mathrm{LHA}\text{\_}P}^{\mathrm{Min}}〉}_{i}^{X}$. Its value represents the average interaction distance between the LHA of atom type X and the closest protein heavy atom. The *g*_O_(***r***_LHA_) and ${〈r_{\mathrm{LHA}\text{\_}P}^{\mathrm{Min}}〉}_{i}^{X}$ values for each point are labeled in Figure 7. For all types of ligand atoms, ${〈r_{\mathrm{LHA}\text{\_}P}^{\mathrm{Min}}〉}_{i}^{X}$ decreased as *g*_O_(***r***_LHA_) was increased. The interaction partners of the protein for each point are also classified in the pie charts of Figure 7. For all types of atoms in the ligand, except for SO_2_ sulfur (S.o2), the ratios of oxygen and nitrogen significantly increased, especially when the interaction distance was within that of hydrogen bonding (3.2 Å). Another observation for the interaction partner elements with all ligand atom types is that the ratio of carbon increased as *g*_O_(***r***_LHA_) was decreased. These observations reflect two facts. (a) The three‐dimensional structure of the protein binding site determines the hydration state of water molecules there. (b) The hydration state of the water molecules determines which element in the ligand is likely to replace them.

Below, we present a detailed analysis for each ligand atom type. Nitrogen, which includes N.2, N.4, amide nitrogen (N.am), N.ar, and N.pl3, is discussed first. For points having an interaction distance ${〈r_{\mathrm{LHA}\text{\_}P}^{\mathrm{Min}}〉}_{i}^{X}$ smaller than the hydrogen bonding distance (3.2 Å), the nearest‐neighbor protein heavy atom was most likely nitrogen or oxygen. In the cases of N.2 (point 3: *g*_O_(***r***_LHA_) = 5.55, ${〈r_{\mathrm{LHA}\text{\_}P}^{\mathrm{Min}}〉}_{3}^{N.2}\;$= 3.11 Å) and N.ar (point 3: *g*_O_(***r***_LHA_) = 7.35, ${〈r_{\mathrm{LHA}\text{\_}P}^{\mathrm{Min}}〉}_{3}^{N.\mathrm{ar}}\;$= 3.01 Å), which have a lone pair and function as hydrogen bond acceptors, the ratio of nitrogen that could act as a hydrogen bond donor in the form of NH increased. For the cases of N.am (point 3: *g*_O_(***r***_LHA_) = 4.65, ${〈r_{\mathrm{LHA}\text{\_}P}^{\mathrm{Min}}〉}_{3}^{N.\mathrm{am}}\;$= 3.01 Å), N.4 (point 2: *g*_O_(***r***_LHA_) = 2.85, ${〈r_{\mathrm{LHA}\text{\_}P}^{\mathrm{Min}}〉}_{2}^{N.4}$ = 3.15 Å; point 3: *g*_O_(***r***_LHA_) = 4.35, ${〈r_{\mathrm{LHA}\text{\_}P}^{\mathrm{Min}}〉}_{3}^{N.4}$ = 3.00 Å; point 4: *g*_O_(***r***_LHA_) = 7.05, ${〈r_{\mathrm{LHA}\text{\_}P}^{\mathrm{Min}}〉}_{4}^{N.4}\;$= 2.73 Å), and N.pl3 (point 3: *g*_O_(***r***_LHA_) = 5.25, ${〈r_{\mathrm{LHA}\text{\_}P}^{\mathrm{Min}}〉}_{3}^{N.\mathrm{pl}3}$ = 2.73 Å; point 4: *g*_O_(***r***_LHA_) = 8.25, ${〈r_{\mathrm{LHA}\text{\_}P}^{\mathrm{Min}}〉}_{4}^{N.\mathrm{pl}3}\;$= 2.82 Å), a majority of the binding partners of the protein were oxygen. The N.am, N.4, and N.pl3 atoms would be hydrogen bond donors in the form of NH because almost all these nitrogen atoms have at least one bound hydrogen atom. These observations suggested that as *g*_O_(***r***_LHA_) increases, the hydrogen bonding interaction between the ligand and the protein becomes dominant, and the interaction distance becomes shorter.

Next, we consider oxygen, which includes O.2 and O.3 (sp2 and sp3 oxygen, respectively). For point 3 of O.2 (*g*_O_(***r***_LHA_) = 7.95, ${〈r_{\mathrm{LHA}\text{\_}P}^{\mathrm{Min}}〉}_{3}^{O.2}$ = 2.97 Å), which has two lone pairs and acts as a hydrogen bond acceptor, most of the binding partners were nitrogen. In contrast, for point 3 of O.3 (*g*_O_(***r***_LHA_) = 7.95, ${〈r_{\mathrm{LHA}\text{\_}P}^{\mathrm{Min}}〉}_{3}^{O.3}$= 2.77 Å), which could act as both a hydrogen bond donor and acceptor in the form of OH, the major binding partners were nitrogen and oxygen. Interestingly, the interaction distance for point 3 of O.2 (2.97 Å) is slightly longer than that of O.3 (2.77 Å). This might be due to the fact that the hydrogen‐bonding distance of the NH—O (protein NH–ligand O.2) interaction is slightly longer than that of OH—O (ligand OH [O.3]–protein O).^28^

Regarding the neutral carbon atoms of the ligand, which include C.2, C.3, and C.ar (sp2, sp3, and aromatic carbon, respectively), no peak with an interaction distance ${〈r_{\mathrm{LHA}\text{\_}P}^{\mathrm{Min}}〉}_{i}^{X}$ smaller than the hydrogen bonding distance (3.2 Å) was observed. This was also true for the points in the highly hydrated region in Figure 6 (point 3 of C.2: *g*_O_(***r***_LHA_) = 6.45, ${〈r_{\mathrm{LHA}\text{\_}P}^{\mathrm{Min}}〉}_{3}^{C.2}$ = 3.36 Å; point 4 of C.3: *g*_O_(***r***_LHA_) = 5.85, ${〈r_{\mathrm{LHA}\text{\_}P}^{\mathrm{Min}}〉}_{4}^{C.3}$= 3.38 Å; and point 3 of C.ar: *g*_O_(***r***_LHA_) = 7.35, ${〈r_{\mathrm{LHA}\text{\_}P}^{\mathrm{Min}}〉}_{3}^{C.\mathrm{ar}}\;$= 3.32 Å), and a majority of the binding partners were oxygen. This strongly suggested the formation of a CH—O (ligand CH–protein O) interaction.

For cationic carbon (C.cat), which could be the central carbon of amidine (R‐C^+^[NH_2_]NH_2_) or guanidine (R‐NHC^+^[NH_2_]NH_2_), as an example, when we look at point 4 (*g*_O_(***r***_LHA_) = 7.95, ${〈r_{\mathrm{LHA}\text{\_}P}^{\mathrm{Min}}〉}_{4}^{C.\mathrm{cat}}\;$= 3.17 Å), the binding partner on the protein side was oxygen. This is clearly due to a strong ionic interaction between a C.cat atom of the ligand and the anionic oxygen of the protein.

**FIGURE 8:**
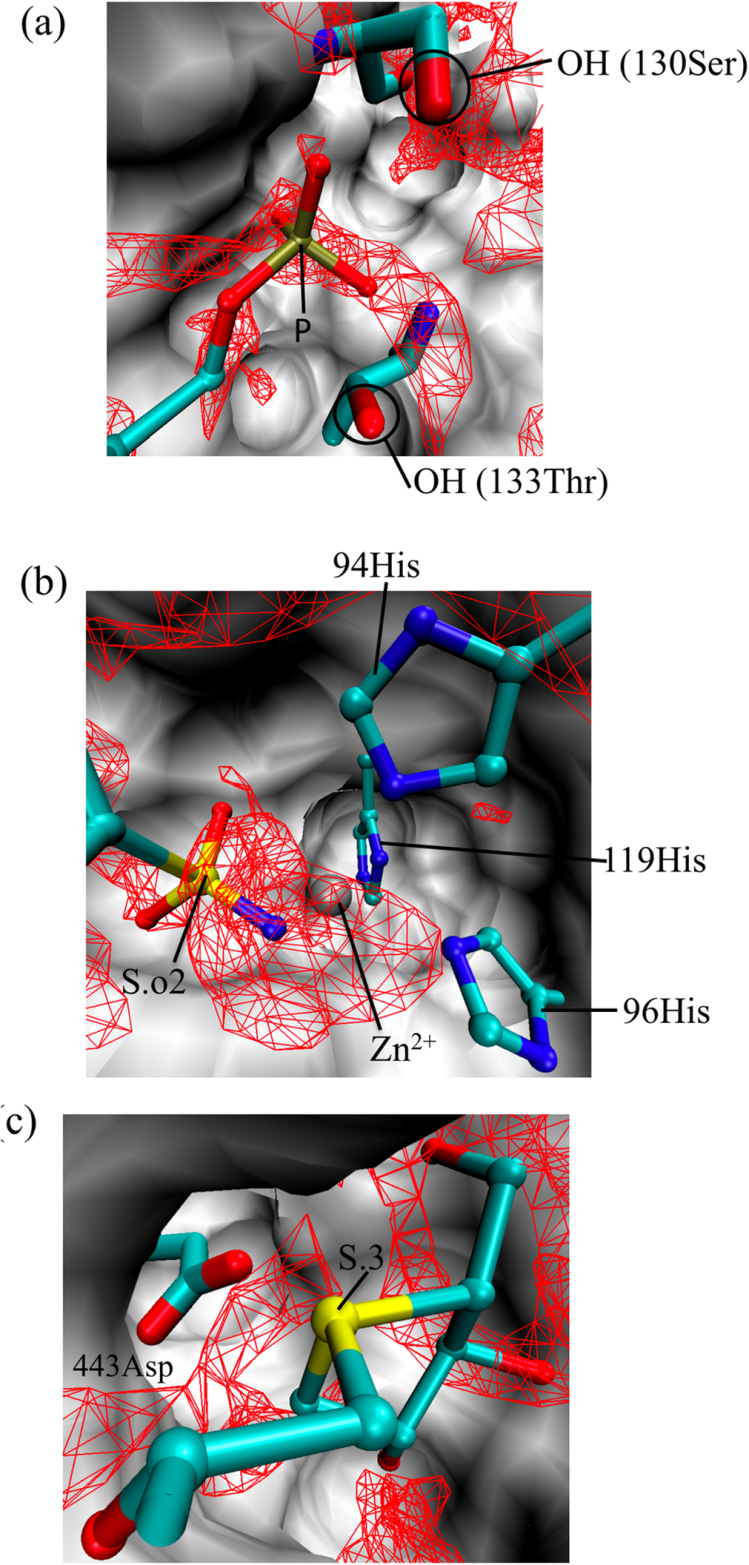
Three‐dimensional distribution functions of the oxygen site of water, *g*_O_(***r***), around (a) *Giardia* GPRTase (PDB code: 1dqn, ligand ID: IMU), (b) carbonic anhydrase II (PDB code: 1g52, ligand ID: F2B), and (c) N‐terminal human maltase‐glucoamylase (PDB code: 3l4u, ligand ID: DSK). The ligand in the correct pose are shown in ball and stick representation. Red mesh: regions with (a) *g*_O_(***r***) > 3.5 and (b, c) *g*_O_(***r***) > 4.0. Surface: protein. The residues are shown in licorice representation. In (a), Gly131 and His132 are ignored in order to clearly depict the distribution function and the ligand [Color figure can be viewed at wileyonlinelibrary.com]

For P.3, which often appears at the center of phosphate (R‐PO_4_^3−^), when we look at point 3 (*g*_O_(***r***_LHA_) = 6.15, ${〈r_{\mathrm{LHA}\text{\_}P}^{\mathrm{Min}}〉}_{3}^{P.3}\;$= 3.36 Å), the major nearest‐neighbor protein atom of the protein was oxygen. This phenomenon cannot be explained by a direct interaction between the protein oxygen and a P.3 atom of the ligand. This phenomenon occurs because of an interaction between the protein oxygen and atoms adjacent to and bonded to the ligand phosphorus atom (typically oxygen). An example is shown in Figure 8a. The P.3 atom is close (3.4 Å) to the protein oxygen because two anionic oxygen atoms of the phosphate group of the ligand form two hydrogen bonds with the hydroxy group on the side chain of Ser130. For this ligand binding process, the water molecules located in the highly hydrated regions around Ser130 (red mesh) are thought to be replaced by the phosphate group of the ligand.

For sulfur, sulfone sulfur (S.o2) and sp3 sulfur (S.3) should be discussed separately because their nearest‐neighbor atoms were different (Figure 7). In the case of S.o2, the nearest‐neighbor atoms of point 3 (*g*_O_(***r***_LHA_) = 19.05, ${〈r_{\mathrm{LHA}\text{\_}P}^{\mathrm{Min}}〉}_{3}^{S.o2}\;$= 2.53 Å) were all Zn^2+^. Similar to the case of P.3, this phenomenon cannot be explained by a direct interaction between Zn^2+^ and the sulfur atom of the ligand. An example is shown in Figure 8b. In this case, the nitrogen and oxygen atoms were next to and covalently bonded to the sulfur atom, respectively, and coordinated to Zn^2+^. This coordination causes the S.o2 atom to be in very close proximity to Zn^2+^. For the corresponding ligand binding process, the water molecules coordinated to Zn^2+^ in the highly hydrated regions (red mesh) are thought to be replaced by the sulfonamide group of the ligand.

For S.3, the interaction partners of point 3 (*g*_O_(***r***_LHA_) = 6.45, ${〈r_{\mathrm{LHA}\text{\_}P}^{\mathrm{Min}}〉}_{3}^{S.3}\;$= 3.26 Å) consisted of nitrogen, oxygen, and carbon in almost equal proportions. An example with oxygen as the interaction partner is shown in Figure 8c. In this case, a S—O interaction was observed between the S.3 atom of the ligand and the oxygen on the side chain of Asp443.^29^

Regarding F, the interaction partners of point 3 (*g*_O_(***r***_LHA_) = 7.35, ${〈r_{\mathrm{LHA}\text{\_}P}^{\mathrm{Min}}〉}_{3}^{F}$ = 3.17 Å) were most likely carbon, followed by oxygen and nitrogen. The origins of these interactions are believed to be the CH‐F interaction, orthogonal multipolar CF—O=C interaction,^30^ and NH—F interaction, respectively.

In the case of Cl, the major interaction partner of point 3 (*g*_O_(***r***_LHA_) = 7.35, ${〈r_{\mathrm{LHA}\text{\_}P}^{\mathrm{Min}}〉}_{3}^{\mathrm{Cl}}\;$= 3.32 Å) was oxygen. One possible origin of this interaction is a halogen bond between the Cl of the ligand and an oxygen of the protein, typically a carbonyl oxygen (O=C). The second major interaction partner of point 3 was carbon, which could be due to a Cl—π interaction between the Cl of the ligand and the aromatic rings of the protein.^31^

For all the points with ${〈r_{\mathrm{LHA}\text{\_}P}^{\mathrm{Min}}〉}_{i}^{X}$ < 4 Å and all atom types, a key observation was that the ratio of carbon in the nearest‐neighbor atoms increased as *g*_O_(***r***_LHA_) decreased. A small *g*_O_(***r***_LHA_) value means a moderately hydrated state, and hence fewer water molecules are replaced upon ligand binding. When the LHA is neutral carbon (C.2, C.3, and C.ar), this phenomenon is intuitively reasonable because the frequency of a hydrophobic interaction between carbon atoms is expected to increase as the binding site becomes more hydrophobic (i.e., less hydrated). On the other hand, careful consideration is required when the LHA is a polar heteroatom such as nitrogen or oxygen. From the viewpoint of entropy, ligand binding is accompanied by an increase in water entropy, primarily due to the EV effect.^32^ From the viewpoint of enthalpy, a desolvation energy, which mainly consists of an electrostatic term and a van der Waals interaction term, is required when water molecules leave the protein. This energy loss should be compensated by favorable interactions between the ligand and the protein and between water molecules, and by the water entropy gain. The less hydrated the binding site, which corresponds to a smaller *g*_O_(***r***_LHA_) value, the fewer water molecules there are to be replaced and the smaller the desolvation energy. Thus, a strong electrostatic interaction (e.g., the strong hydrogen bond of NH‐O=C) is not required and weak interactions such as CH—O and CH—F are sufficient if the desolvation energy to be compensated is small. This might be a reason why the ratio of carbon increased when *g*_O_(***r***_LHA_) was small.

Using the results in Figures 6 and 7, we propose the following picture of ligand binding. In highly hydrated regions, P(*g*_O_(***r***_LHA_)) was higher for the correct poses and lower for the incorrect ones. It was also observed that *g*_O_(***r***) was generally larger at positions closer to the protein surface. Thus, the correct binding pose might be the one that maximizes the occupation of highly hydrated regions very close to the protein. A possible way to achieve such maximum occupation is a binding pose with tight contacts with the protein. This picture is consistent with the proposal by Kinoshita that in biological self‐assembly processes such as protein folding and ligand binding, a tightly packed conformation occurs to maximize the translational entropy of water.^32^ The other way is to properly position polar heteroatoms of the ligand to maximize the occupation of highly hydrated regions. The correct binding pose is thought to be characterized simultaneously by balanced tight packing and a proper allocation of the polar heteroatoms.

## 4 CONCLUSIONS

In order to comprehensively examine the hydration states of ligand binding sites in proteins, the distribution functions for the oxygen of water, *g*_O_(***r***), were calculated for the ligand‐free structures of 3,706 proteins in the PDBbind refined set (v. 2017) using the 3D‐RISM theory. For approximately 620,000 CWs close to the proteins, the maximum value of *g*_O_(***r***_CW_) exceeded 30, and the peak of the probability distribution of *g*_O_(***r***_CW_) was around 5.

A comparison of the probability distribution of *g*_O_(***r***_CW_) between all CWs and those close to the ligand revealed that the latter are more likely to be in a moderately hydrated state. Among the CWs close to the ligand, those in a highly hydrated state primarily interact with oxygen and nitrogen in the proteins from a hydrogen‐bonding distance, while those in a moderately hydrated state predominantly interact with carbon from beyond the hydrogen‐bonding distance. In two examples, the formation of ligand/CW/protein interaction networks was observed.

Regarding the hydration state of the LHAs, when the LHA was a heteroatom, the peak height in the distribution of *g*_O_(***r***_LHA_) at high *g*_O_(***r***_LHA_) values, e.g.,∼8, was higher for the correct poses, whereas that at low *g*_O_(***r***_LHA_) values, e.g.,∼1, was higher for the incorrect poses. These observations suggested that the correct pose may be distinguished from incorrect ones by examining the overlap between the polar heteroatoms of the ligand and the highly hydrated regions (i.e., with high *g*_O_(***r***_LHA_) values) of the binding site. When the distance between the LHAs in proteins and their interaction partners ($r_{\mathrm{LHA}\text{\_}P}^{\mathrm{Min}}$) was within the distance of a hydrogen bond, *g*_O_(***r***_LHA_) mostly ranged from 7 to 8 and their partners were oxygen or nitrogen in the proteins. Hence, the protein environment (in this case, the oxygen and nitrogen atoms at the protein surface) seems to determine the hydration states around the protein and facilitates the formation of hydrogen bonds. In contrast, for 3.2 Å $\leq r_{\mathrm{LHA}\text{\_}P}^{\mathrm{Min}}\leq$ 4 Å, the *g*_O_(***r***_LHA_) values ranged mostly from 3 to 6 and the major interaction partner was carbon. Therefore, compared with oxygen and nitrogen atoms, carbon atoms at the protein surface make the surface less hydrated. Consequently, the protein surface is more suitable for weak interactions such as CH—O, CH—F, CH—π, and hydrophobic interactions.

It is suggested that binding‐pose prediction would be possible based on the following results. Water molecules in highly hydrated regions are likely to be replaced by the polar heteroatoms of the LHAs. However, the water molecules in moderately hydrated regions are more likely to be replaced by the hydrophobic atoms of the LHAs than by the polar heteroatoms of the LHAs. In the WaterMap method, unfavorable hydration sites are amenable to binding.^4^ This idea is consistent with our result that water molecules in moderately hydrated regions are more likely to be replaced by the hydrophobic atoms of the LHAs.

One remaining issue is to elucidate the effects of the structural dynamics of proteins on their hydration state. In the present study, we analyzed the hydration states of ligand binding sites with the assumption that no structural change occurred upon ligand binding. This is because *apo* structures were not always available, and thus it is unclear how the protein conformation changes upon ligand binding. In future, an analysis of the hydration states must be performed with the structural changes of proteins upon ligand binding taken into consideration using MD simulations. A bottleneck for this analysis would be that comprehensive calculations of the hydration states for multiple conformations (e.g., generated by MD simulations) of a large number of proteins still requires huge computation time. Thus, the speed of hydration state computation using the 3D‐RISM theory must be improved. Another issue is the force field. While the Amber ff99SB force field and the coincident SPC/E water model were found to be reasonable in this analysis, the quality of force field calculations is not comparable to that of quantum mechanics calculations. The adoption of other currently available force fields could be helpful, and more accurate new ones may have to be devised in the future.

The analysis in this study can be extended to other systems such as protein–protein complexes or mixtures of water and a fragment of a drug molecule.^33^ The hydration state at the protein–protein interface would be different from that at the protein‐ligand interface because the shape and properties of the interfaces are different. For instance, while the ligand binding site is a groove or a deep pocket in the protein, the protein–protein interface is often flat. Thus, water may play different roles in the formation of these two types of protein complexes. Extending the present method to protein–protein complexes would shed light on the roles of water in their formation.

For the mixtures of water and a fragment of a drug molecule, the solvation state of the fragment at the protein binding site, which was obtained using MD simulations of a protein in a mixture of water and the fragment, has been used for virtual screening^34^ and the identification of hot spots on the protein surface.^35^ However, only a few proteins have been considered for such applications. Using the 3D‐RISM theory to analyze the solvation state of the fragment at the binding sites would allow a comprehensive characterization of the hot spots for thousands of proteins. These extensions will be reported in further publications.

## Supporting information


**Appendix** S1. Supporting Information
