# Supplementary material for: Comprehensive 3D‐RISM analysis of the hydration of small molecule binding sites in ligand‐free protein structures
Source: J Comput Chem. 2020 Aug 19;41(28):2406–19. doi: 10.1002/jcc.26406 (PMC7540010; doi:10.1002/jcc.26406)
Supplement: Supplementary file 1 — Appendix S1. Supporting Information [file JCC-41-2406-s001.pdf]

Supporting Information

Comprehensive Analysis of the Hydration of Small Molecule Binding Sites in Ligand-Free Protein Structures: 3D-RISM Approach

Takashi Yoshidome[*,†], *Mitsunori Ikeguchi*[‡,§], *and Masateru Ohta*[*,‡]

[†]*Department of Applied Physics, Graduate School of Engineering, Tohoku University, Sendai 980-8579, Japan*

[‡]*Drug Development Data Intelligence Platform Group, Medical Science Innovation Hub Program, Cluster of Science, Technology and Innovation Hub, RIKEN, 1-7-29, Suehiro-cho, Tsurumi-ku, Yokohama 230-0045, Japan*

[§]*Graduate School of Medical Life Science, Yokohama City University, 1-7-29, Suehiro-cho, Tsurumi-ku, Yokohama 230-0045, Japan*

[*]Corresponding Authors:

Takashi Yoshidome (Email: t4yoshidome@camp.apph.tohoku.ac.jp)

Masateru Ohta (Email: masateru.ota@riken.jp)

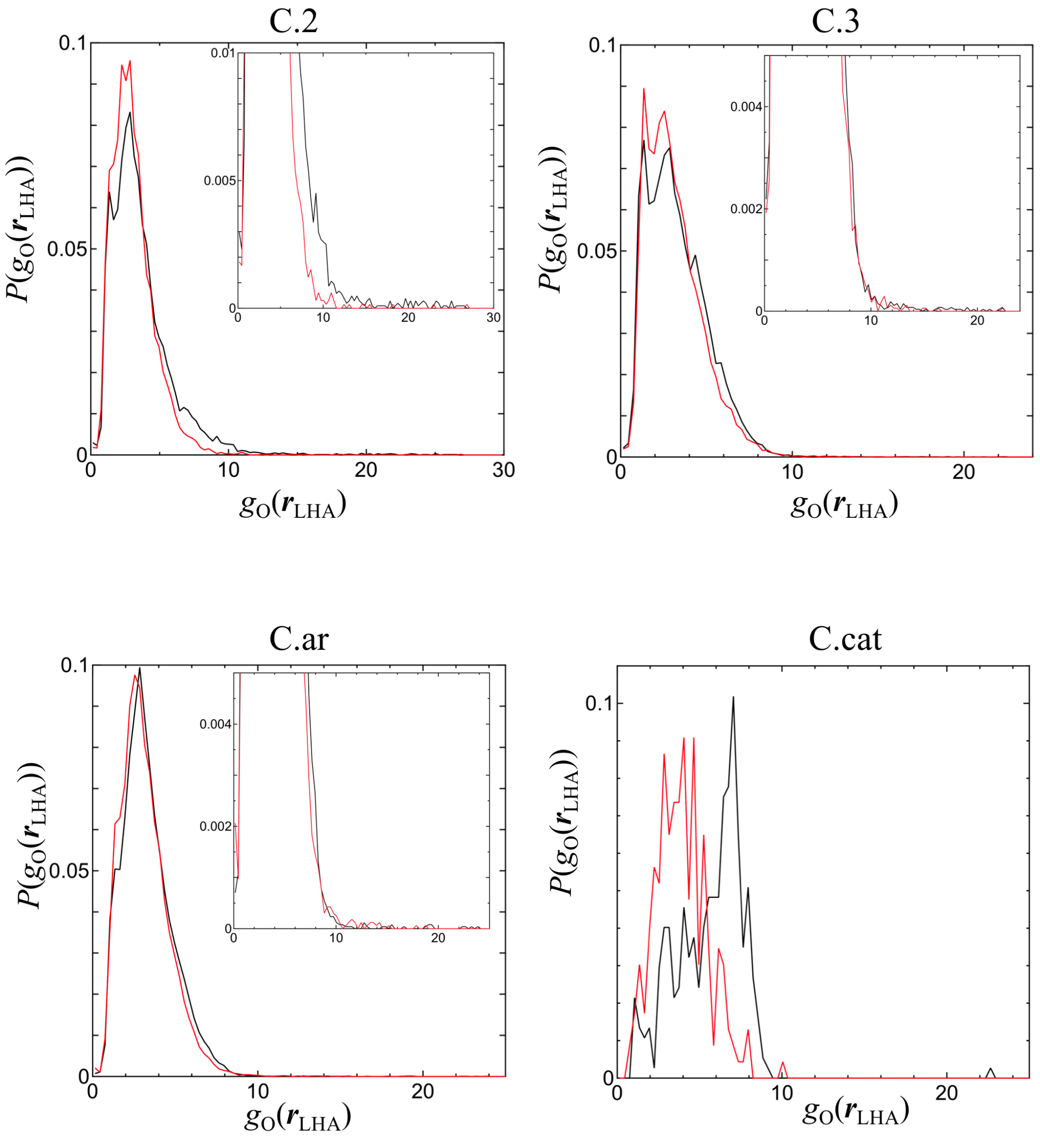

Figure. S1 Distribution plots of the probability of $g_O(\boldsymbol{r}_{LHA})$ for each type of ligand atom. Here, the distribution plots in Figure 6 of the main text are depicted in the full range of $g_O(\boldsymbol{r}_{LHA})$. Black and red lines are results for the correct and incorrect poses, respectively. The insets are enlarged views for the low $P\left(g_O(\boldsymbol{r}_{LHA})\right)$ range. If the full range for the probabilities of $g_O(\boldsymbol{r}_{LHA})$ is already shown in Figure 6, then the corresponding plots are not illustrated here.

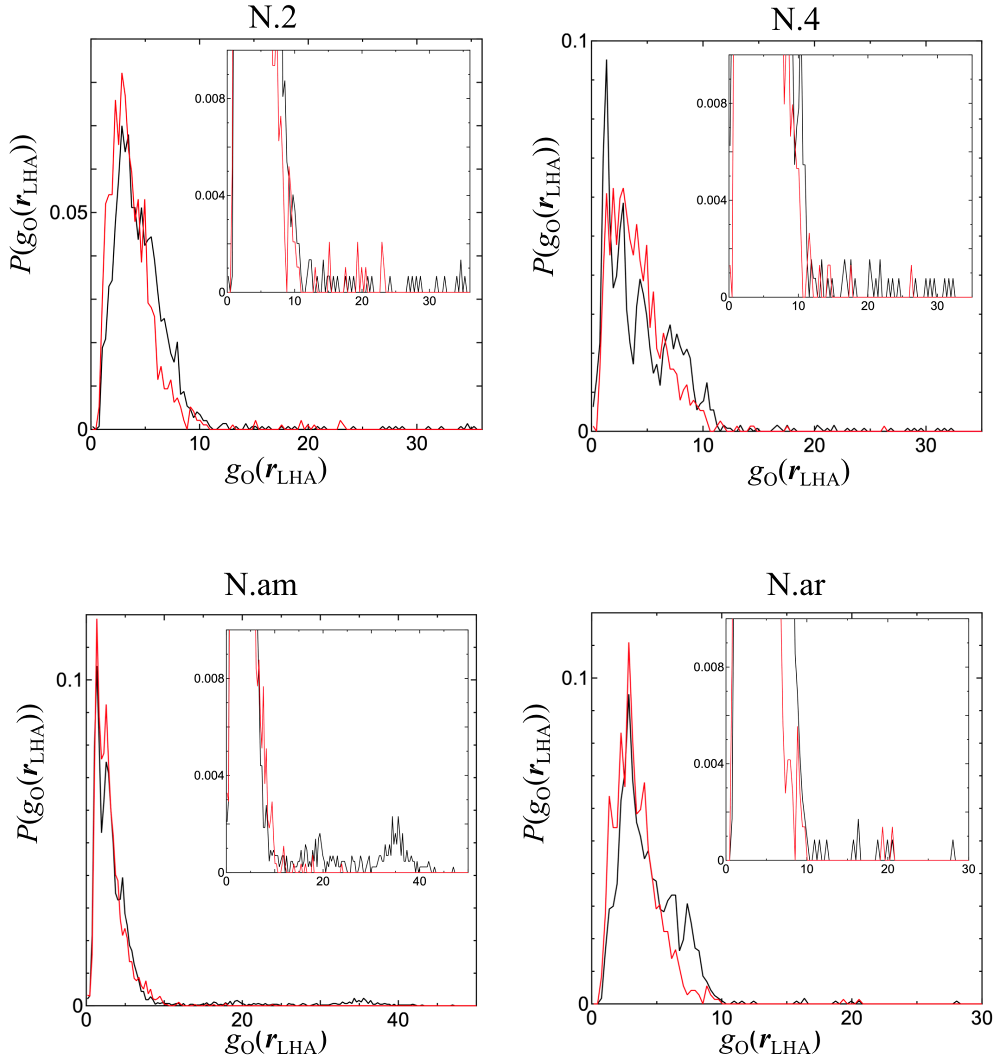

Figure. S1 (Continued)

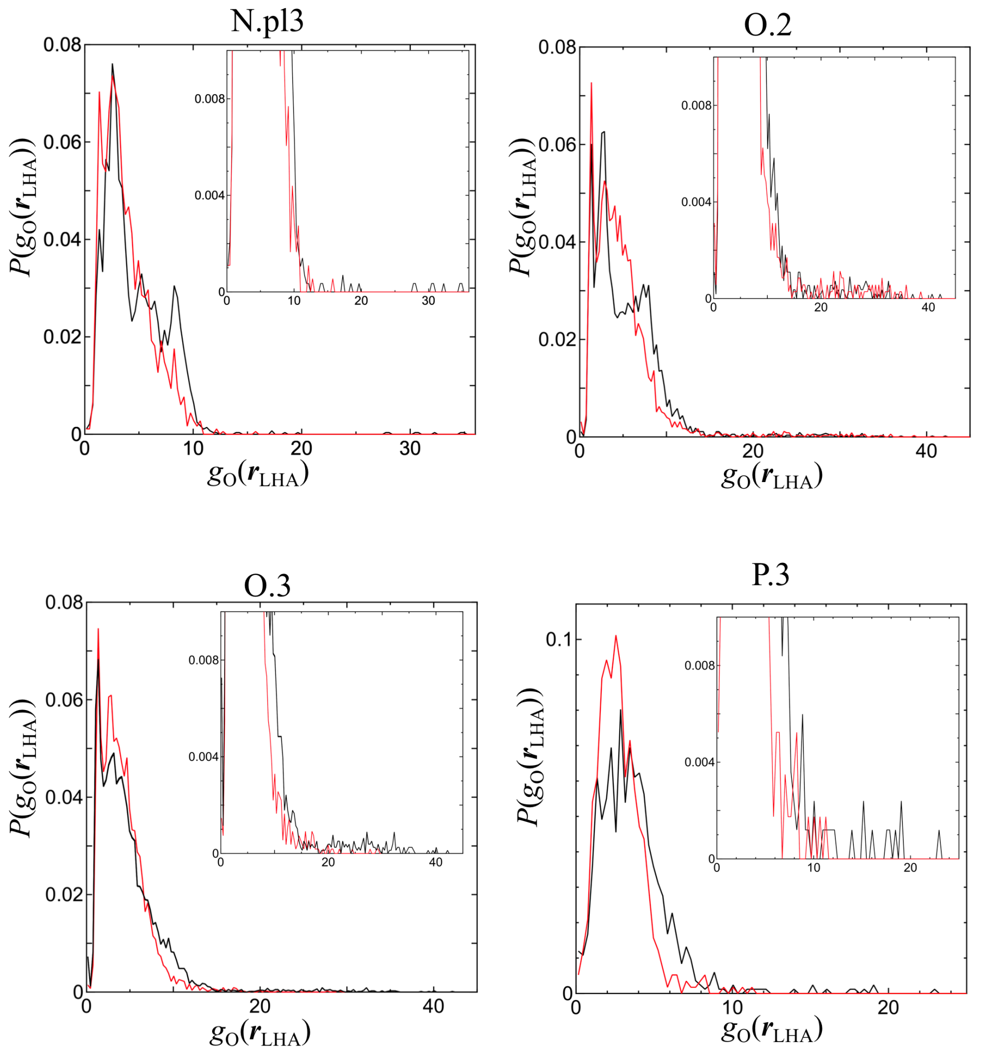

Fig. S1 (Continued)

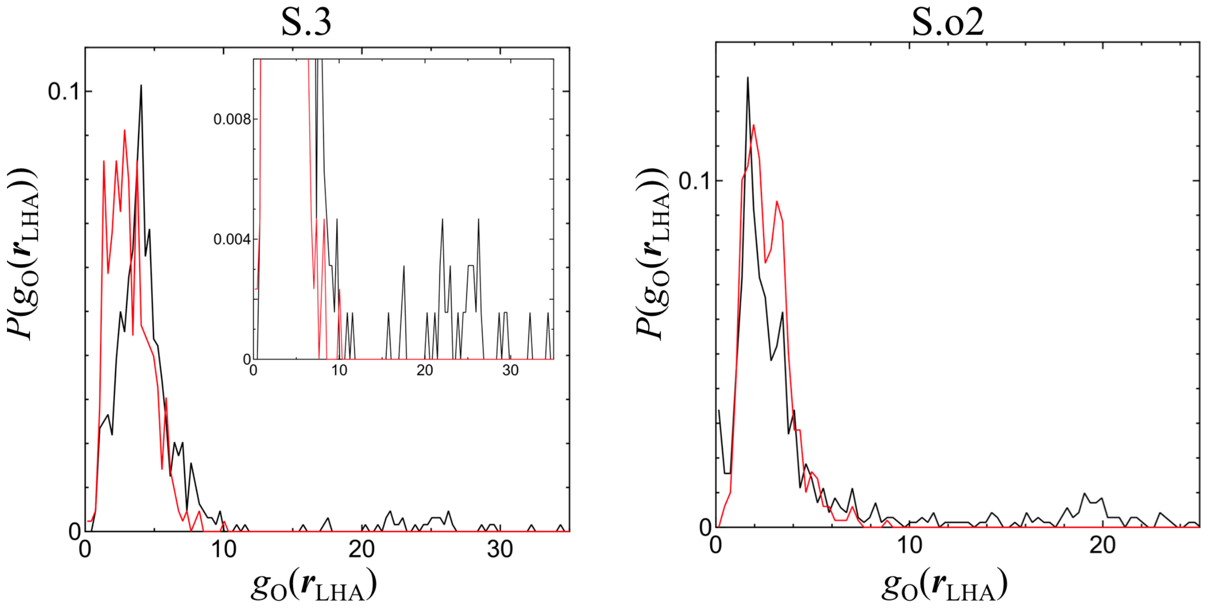

Fig. S1 (Continued)
